# Severe Burn from Vaginal Douche

**Published:** 1907-02

**Authors:** C. Lambert

**Affiliations:** New York City


### SEVERE BURN FROM VAGINAL DOUCHE.

By C. Lambert, M. D., New York City.

The case I present I consider of special significance, emphasizing as it does, the danger attendant upon the use of toxic and irritating solutions, especially in thoughtless and careless hands and also demonstrating the value of a solution of a non-toxic and non-irritant nature, such as has been brought to a state of perfection in Glyco-Thymoline.

I was hurriedly called to see Mrs. H., age 22, whom I found suffering from a most severe burn involving the vaginal mucous membrane, the epithelium of which was completely denuded, while the perineum and adjacent parts had also suffered quite a loss of epidermis. The destructive process in the vaginal tract extended throught the superficial fascia and was quite painful. She had, it seems, obtained a curbstone prescription from a medical acquaintance whom she met on the street a few days previous. Her mind, however, became confused and the proportions as was directed, i. e., one dram to two quarts of water, were just the reverse of what she did use, i e., two drams to one quart of water, resulting when applied as stated above.

I at once administered a vaginal douche composed of two ounces of Glyco-Thymoline to two pints of tepid water, after which I applied a layer of cotton or loose tampon saturated with pure Glyco-Thymoline within the vagina and a dressing of the same was applied to the perineum, etc. The tampon I did not renew until forty-one hours later. A call out of the city, at which I was detained, prevented me from removing it at the end of twenty-four hours as I had intended to do, and it was with no little misgiving that I hastened to give it my attention. It was, however, only another exhibition of one of Glyco-Thymoline's most valuable properties, as the cotton was as sweet and clean as regards all odor, etc., as when applied, and since then I have frequently assured myself that the preventing of decomposition of the discharges is a most valuable attribute of Glyco-Thymoline. The same dressing, vaginal and otherwise, was repeated at this visit, and at the next I found such decided improvement, the tissues in so healthy a state, that I merely ordered a continuance of the vaginal douche twice a day of Glyco-Thymoline, one ounce to one pint of water, which treatment, at the end of another week, affected a cure.

---

On December 9 there was only one case of yellow fever reported under treatment in Havana and one in the provinces. During the week ended December 17 two new cases were reported in Havana and one in the provinces, with no deaths. At this time last year there were 16 cases in Havana.

---

The Nobel prize in Medicine for this year, which amounts to approximately $40,000, has been equally divided between Professor Golgi, for his method of staining brain tissues, and Professor Ramon Y. Cajal for his developments in nerve and brain anatomy, and particularly for the application of Golgi's discovery to research in embryonic tissue.

---

Jacob Tararosky, Brooklyn, a student at the College of Physicians in the City of New York, is reported to have killed himself by taking carbolic acid, December 3. He had made a special study of tuberculosis and, it is said, became inoculated with the disease.

---

The Hospital for Crippled and Deformed Children netted $1,500 as the proceeds of a recent vaudeville entertainment at the Maryland Theater.

---

Dr. Maverell K. Allen, health officer of Louisville, threatens to issue warrants for the arrest of physicians who fail to report cases of tuberculosis to the health officer, as required by the city ordinance.

---

L. W. Bremerman, A.M., M.D., of New York City, has been appointed Professor of Genito-Urinary Diseases in the New York School of Clinical Medicine to fill the vacancy caused by the death of Professor William K. Otis, M.D.

